# Genetic Susceptibility to Refractive Error: Association of Vasoactive Intestinal Peptide Receptor 2 (*VIPR2*) with High Myopia in Chinese

**DOI:** 10.1371/journal.pone.0061805

**Published:** 2013-04-18

**Authors:** Wai Chi Yiu, Maurice K. H. Yap, Wai Yan Fung, Po Wah Ng, Shea Ping Yip

**Affiliations:** 1 Centre for Myopia Research, School of Optometry, The Hong Kong Polytechnic University, Hong Kong SAR, China; 2 Department of Health Technology and Informatics, The Hong Kong Polytechnic University, Hong Kong SAR, China; Kunming Institute of Zoology, Chinese Academy of Sciences, China

## Abstract

Myopia is the most common ocular disease worldwide. We investigated the association of high myopia with the common single nucleotide polymorphisms (SNPs) of five candidate genes – early growth response 1 (*EGR1*), v-fos FBJ murine osteosarcoma viral oncogene homolog (*FOS*), jun oncogene (*JUN*), vasoactive intestinal peptide (*VIP*), and vasoactive intestinal peptide receptor 2 (*VIPR2*). We recruited 1200 unrelated Chinese subjects with 600 cases (spherical equivalent ≤−8.00 diopters) and 600 controls (spherical equivalent within ±1.00 diopter). A discovery sample set was formed from 300 cases and 300 controls, and a replication sample set from the remaining samples. Tag SNPs were genotyped for the discovery sample set, and the most significant haplotypes and their constituent SNPs were followed up with the replication sample set. The allele and haplotype frequencies in cases and controls were compared by logistic regression adjusted for sex and age to give *P*_a_ values, and multiple comparisons were corrected by permutation test to give *P*_aemp_ values. Odd ratios (OR) were calculated accordingly. In the discovery phase, *EGR1*, *JUN* and *VIP* did not show any significant association while *FOS* and *VIPR2* demonstrated significant haplotype association with high myopia. In the replication phase, the haplotype association for *VIPR2* was successfully replicated, but not *FOS*. In analysis combining both sample sets, the most significant association signals of *VIPR2* were the single marker rs2071625 (*P*_a_ = 0.0008, *P*_aemp_ = 0.0046 and OR = 0.75) and the 4-SNP haplotype window rs2071623-rs2071625-rs2730220-rs885863 (omnibus test, *P*_a_ = 9.10e-10 and *P*_aemp_ = 0.0001) with one protective haplotype (GGGG: *P*_aemp_ = 0.0002 and OR = 0.52) and one high-risk haplotype (GAGA: *P*_aemp_ = 0.0027 and OR = 4.68). This 4-SNP haplotype window was the most significant in all sample sets examined. This is the first study to suggest a role of *VIPR2* in the genetic susceptibility to high myopia. *EGR1*, *JUN*, *FOS* and *VIP* are unlikely to be important in predisposing humans to high myopia.

## Introduction

Myopia is the most common eye disorder worldwide and has reached epidemic prevalence in East Asia [1]. Myopia can be classified into different categories based on the clinical entity [2]. High myopia is defined as a refractive error equal to or worse than −6.00 diopters (D). It is the most concerned type because of its association with irreversible visual impairment such as retinal detachment, glaucoma and, in severe cases, blindness [3]. Although both environmental and genetic factors play important roles in the development of myopia [4], [5], the principal factor is still under debate. Cross-sectional studies have shown that environmental factors such as educational level and near work are associated with the development of myopia [1], [6]. Evidence pointing to the role of genetic factors in the etiology of myopia comes mainly from the identification of myopia loci/genes in linkage and/or association studies [7]–[15]. Myopia is a complex disease, and genetic variations can increase the susceptibility to environmental factors and cause an early onset and/or aggressive progression. As the age of myopia onset is decreasing [16], [17], the chance of developing high myopia increases. In order to control the progression of myopia, the underlying pathways leading to this condition must be understood.

Animal studies show that the development of myopia involves extracellular matrix (ECM) remodeling of the sclera [18], [19]. Therefore, candidate-gene association studies in myopia genetics have mainly focused on the genes expressed in the sclera [20]–[23]. However, it is well established that visual experience alters ocular growth and the changes are first mediated by local visual processing and signaling mechanisms [24]–[26]. We hypothesize that genes directly responsive to visual signals are the primary genes involved in the biological pathways. Activation of these genes may further activate other secondary genes in the pathways, and this in turn causes ECM remodeling of the sclera and ultimately leads to altered ocular growth. This study aims to investigate these primary genes for their potential role in the susceptibility to high myopia. Five functional candidate genes have been selected on the basis of this hypothesis: early growth response 1 (*EGR1*) located at chromosome 5q31.1, v-fos FBJ murine osteosarcoma viral oncogene homolog (*FOS*) at 14q24.3, jun oncogene (*JUN*) at 1p32-p31, vasoactive intestinal peptide (*VIP*) at 6q25, vasoactive intestinal peptide receptor 2 (*VIPR2*) at 7q36.3 (NCBI, http://www.ncbi.nlm.nih.gov/).

Consistent findings from different animal models demonstrate the participation of Egr-1 (also known as ZENK) in ocular growth [27]–[29]. The study of *Egr-1* knockout mice has also provided convincing evidence for the involvement of *Egr-1* in regulating ocular growth [30]. *Egr-1* knockout mice had a myopic shift in refraction and tended to have eyes with a longer axial length. *FOS* encodes a protein that dimerizes with the protein encoded by *JUN* to form a transcription factor complex known as activating protein-1 (AP-1) [31]. Binding of AP-1 causes trans-activation of its target genes. AP-1 sites were found in the promoters of genes encoding matrix metalloproteinases (*MMPs*) and their inhibitors (tissue inhibitor of metalloproteinases or *TIMPs*) [32], [33]. In other words, AP-1 can regulate the expression of *MMPs* and *TIMPs*. MMPs are capable of degrading ECM proteins and are believed to be involved in ECM remodeling of the sclera. AP-1 can also repress the trans-activation of retinoid receptors [34], which have been shown to be critical for the regulation of eye growth [35]. The expression of *VIP* showed a positive correlation with the depth of the vitreous chamber [36] and this suggests that increased release of VIP may be responsible for ocular growth. *VIPR2* is one of the *VIP* receptors and is located on chromosome 7q36, which is within the interval for a putative locus for autosomal dominant high-grade myopia (formerly called *MYP4*) [37], [38]. The expression of VIPR2 in the retina and the choroid was altered in chicks with form-deprivation myopia [39]. These findings suggest a potential role of *VIPR2* in the development of myopia.

We conducted a case-control genetic association study to investigate the association between high myopia and the five selected candidate genes. Samples from a homogeneous population (southern Han Chinese) were used to minimize the possibility of false positive results due to population stratification. We also validated the initial positive findings with an independent sample set.

## Materials and Methods

### Recruitment of Subjects

We recruited unrelated Han Chinese aged between 18 and 45 years via the Optometry Clinic of The Hong Kong Polytechnic University as described previously [9]–[11]. Cases were subjects with refraction (spherical equivalent or SE) of −8.00 D or worse for both eyes while controls were subjects with SE within ±1.00 D for both eyes. Subjects with ocular disease or genetic disease associated with myopia were excluded from the study. The study was approved by the Human Subjects Ethics Sub-committee, The Hong Kong Polytechnic University, and adhered to the tenets of the Declaration of Helsinki. All participants gave written informed consent. Eye examination included retinoscopy, fundus examination, and measurement of refractive error, corneal power, lens thickness, anterior chamber depth, posterior chamber depth and axial length [9]–[11]. In total, we recruited 600 cases and 600 controls, and sequentially allocated them into two sample sets (the discovery sample set and the replication sample set). Each sample set consisted of 300 cases and 300 controls.

### Selection and genotyping of single nucleotide polymorphisms (SNPs)

Genomic DNA was extracted from the subjects' blood samples [9]. Tag SNPs of the five candidate genes (Table S1) were identified from the HapMap database (release 23a/phase II March 08; http://hapmap.ncbi.nlm.nih.gov/) with the selection criteria of r^2^>0.8 and minor allele frequency (MAF)>0.10 for Han Chinese population by the Tagger software. The 3-kb regions upstream and downstream of the candidate genes were included for SNP selection [11], [23].

We genotyped the SNPs by restriction fragment length polymorphism, unlabelled probe melting curve analysis or primer extension reaction coupled with denaturing high-performance liquid chromatography [9]–[11], [23]. We used direct DNA sequencing of representative samples to confirm all observed genotypes. Details of the genotyping methods are described in Appendix S1. Tag SNPs and their genotyping methods are listed in Table S1 together with the respective restriction enzymes, primers, extension primer, and probes. Tag SNPs showing significant associations in single-marker or haplotype analyses using the discovery sample set were followed up with the replication sample set.

### Statistical analysis

We used PLINK (ver. 1.07; http://pngu.mgh.harvard.edu/~purcell/plink/) as the software tool for association analysis [40]. Genotypes of the controls were tested for Hardy-Weinberg equilibrium (HWE) by exact test with a significance threshold set at *P* = 0.001 [41]. Single-marker (allelic test) and haplotype associations were tested using logistic regression adjusted for sex and age as covariates, and odds ratios (OR) and their 95% confidence intervals (CI) were also calculated accordingly. We performed haplotype analysis using an exhaustive sliding-window approach by testing all possible haplotypes made up of a variable number of constitutive SNPs. For each sliding window, we jointly assessed the significance of the haplotype effects by a single case-control omnibus test with (H – 1) degrees of freedom, where H is the number of haplotypes for the window concerned. For a particular window size with a given gene, we conducted the test for all possible windows of the same size, and shifted one SNP at a time to the 3′ end of the gene. Multiple testing was corrected by generating empirical *P* values based on 10 000 permutations across all SNPs or haplotype windows for a given sample set as appropriate. To increase the power of the study, the genotype data were also analyzed by combining the two sample sets with adjustment for sample set as another covariate in addition to sex and age. *P* values adjusted for the covariates are reported, and indicated as *P*_a_ if not corrected for multiple comparisons or as *P*_aemp_ if corrected for multiple comparisons. A *P*_aemp_<0.05 indicates significant association. Note that the minimum *P*_aemp_ value achievable by 10 000 permutations is 0.0001. Linkage disequilibrium (LD) maps were constructed by Haploview using solid spine of LD as the definition of haplotype blocks [42].

## Results

### Subject demographics

Measurements for all traits were highly correlated between the right and the left eyes, particularly for SE (r = 0.97) and axial length (r = 0.96). Hence, only measurements for the right eye were used for analysis. The characteristics of the subjects in the discovery and the replication sample sets are presented in Table 1. The average SE values were 0.03 D for controls and −10.56 D for cases of the discovery sample set, and 0.10 D for controls and −10.06 D for cases of the replication sample set. Most control subjects had SE between −0.10 D and 0.10 D with about 2.2% of controls in the range of 0.12 D and 0.40 D. Most case subjects had SE between −8.00 D and −15.00 D with 4.8% of cases in the range of less than −15.00 D and −24.00 D. This skewed distribution arose as a result of the criteria used for subject recruitment.

**Table 1:** Characteristics of subjects in the discovery and the replication sample sets*.

	Discovery sample set		Replication sample set	
Characteristics	Controls	Cases	Controls	Cases
Total number	300	300	300	300
Proportion of females, %	55.60	73.33	59.33	69.67
Age (mean ± SD), years†	24.90±6.10	27.75±6.89	33.34±9.52	33.73±9.09
SE (mean ± SD), D	0.03±0.46	−10.56±2.49	0.10±0.56	−10.16±2.31
AL (mean ± SD), mm	23.85±0.82	27.77±1.15	23.72±0.83	27.55±1.15
ACD (mean ± SD), mm	3.62±0.35	3.72±0.32	3.18±0.41	3.34±0.39
PCD (mean ± SD) , mm	16.30±0.95	19.97±1.21	16.19±0.87	19.89±1.19
LT (mean ± SD), mm	3.94±0.55	4.02±0.55	4.34±0.58	4.29±0.51
CP (mean ± SD), D	43.86±1.61	44.91±1.42	44.19±1.50	44.93±1.48

* The ocular measurements are based on the data of the right eyes.

† The data of age are missing in 2 controls and 3 cases of the discovery sample set, and 2 controls and 1 case of the replication sample set.

Abbreviations: SD, standard deviation; SE, spherical equivalent; AL, axial length; ACD, anterior chamber depth; PCD, posterior chamber depth; LT, lens thickness; CP, corneal power; and D, diopter.

There were fewer females in the control group than in the case group for the discovery sample set (55.60% vs 73.33%; chi-squared test, *P* = 9.146×10^−6^) and for the replication sample set (59.33% vs 69.67%; chi-squared test, *P* = 0.0105). The subjects were younger in the control group than in the case group (mean age, 24.90 vs 27.75 years; unpaired t test, *P* = 3.02×10^−7^) for the discovery sample set. Although the mean age was similar in both the control and the case groups (33.34 vs 33.73 years; unpaired t test, *P* = 0.6118) of the replication sample set, we adjusted for sex and age in all subsequent association analyses for all sample sets to maintain consistency across the board.

### Discovery sample set

In total, 26 tag SNPs were selected and genotyped for the discovery sample set: 1 from *ERG1*, 5 from *FOS*, 4 from *JUN*, 3 from *VIP* and 13 from *VIPR2* (Table S1). Table 2 summarizes the genotype data. The control group was in HWE (*P*>0.001) for all 26 tag SNPs examined. Therefore, we included all SNPs for association analysis. In single-marker analysis, six SNPs showed significant nominal *P* values (*P*_a_<0.05): rs4645874 (S04) of *FOS*, rs1407267 (S01) of *VIP*, and rs3828963 (S03), rs6973238 (S06), rs3793227 (S07) and rs2071623 (S10) of *VIPR2*. However, none of them remained significant (*P*_aemp_<0.05) after correction for multiple comparisons across 26 SNPs by permutation test.

**Table 2:** Functional candidate genes: summary of genotype data and single-marker association analysis.

		Sequential	Alleles†	Genotype counts (11/12/22)		Minor allele freq		*P* value	*P*_a_
Gene	SNP	No. *	(1/2)	Cases	Controls	Cases	Controls	(HWE for controls)	(allelic association)
**Discovery sample set**									
*EGR1*	rs11741807	(S01)	G/T	183/107/10	188/100/12	0.2117	0.2067	0.9513	0.8602
*FOS*	rs7101	(S01)	C/T	81/132/87	95/138/67	0.5100	0.4533	0.2464	0.0816
	rs1063169	(S02)	G/T	190/96/14	187/95/18	0.2067	0.2183	0.2667	0.5630
	rs4645869	(S03)	G/A	221/71/8	207/86/7	0.1450	0.1667	0.7724	0.7302
	rs4645874	(S04)	C/T	224/71/5	206/90/4	0.1350	0.1633	0.1324	0.0208
	rs17103109	(S05)	T/G	149/127/24	149/130/21	0.2917	0.2867	0.3896	0.0801
*JUN*	rs2104259	(S01)	C/G	119/120/61	116/134/50	0.4033	0.3900	0.3325	0.5208
	rs2760501	(S02)	T/G	199/86/15	192/91/17	0.1933	0.2083	0.2146	0.5752
	rs1323288	(S03)	A/C	117/139/44	106/142/52	0.3783	0.4100	0.7751	0.4260
	rs997768	(S04)	T/C	71/174/55	81/153/66	0.4733	0.4750	0.8056	0.8680
*VIP*	rs1407267	(S01)	G/T	189/101/10	216/75/9	0.2017	0.1550	0.5332	0.0237
	rs12201030	(S02)	A/G	226/72/2	239/59/2	0.1267	0.1050	0.6791	0.4515
	rs664355	(S03)	C/T	230/63/7	238/54/8	0.1283	0.1167	0.0649	0.3953
*VIPR2*	rs3812311	(S01)	A/G	166/111/23	179/91/30	0.2617	0.2517	0.0015	0.9931
	rs464260	(S02)	A/G	176/118/6	182/112/6	0.2167	0.2067	0.0210	0.7257
	rs3828963	(S03)	A/T	244/54/2	230/61/9	0.0967	0.1317	0.1175	0.0264
	rs3793238	(S04)	G/A	228/65/7	233/60/7	0.1317	0.1233	0.2862	0.7567
	rs399867	(S05)	C/T	150/117/33	167/101/32	0.3050	0.2750	0.0110	0.4522
	rs6973238	(S06)	T/C	202/82/16	174/95/31	0.1900	0.2617	0.0032	0.0060
	rs3793227	(S07)	C/T	240/56/4	220/68/12	0.1067	0.1533	0.0532	0.0152
	rs2540352	(S08)	G/A	195/85/20	202/83/15	0.2083	0.1883	0.1424	0.1436
	rs6950938	(S09)	G/A	178/92/30	184/98/18	0.2533	0.2233	0.3792	0.1786
	rs2071623	(S10)	G/A	148/97/55	126/119/55	0.3450	0.3817	0.0077	0.0402
	rs2071625	(S11)	A/G	158/117/25	135/120/45	0.2783	0.3500	0.0466	0.1585
	rs2730220	(S12)	G/A	249/41/10	245/49/6	0.1017	0.1017	0.1357	0.0678
	rs885863	(S13)	G/A	210/83/7	197/95/8	0.1617	0.1850	0.5261	0.1508
**Replication sample set**									
*FOS*	rs4645869	(S03)	G/A	214/77/9	213/75/9 §	0.1583	0.1566	0.5072	0.9114
	rs4645874	(S04)	C/T	235/57/8	242/55/3	0.1217	0.1017	1.0000	0.2633
	rs17103109	(S05)	T/G	168/114/18	158/117/25	0.2500	0.2783	0.6660	0.2295
*VIPR2*	rs2071623	(S10)	G/A	132/137/31	125/130/45	0.3317	0.3667	0.2632	0.1506
	rs2071625	(S11)	A/G	145/131/24	124/134/42	0.2983	0.3633	0.5349	0.0169
	**rs2730220**	**(S12)** ‡	G/A	259/41/0	232/65/3	0.0683	0.1183	0.7804	**0.0017** ‡
	rs885863	(S13)	G/A	186/95/19	202/91/7	0.2217	0.1750	0.5470	0.0426
**Combined sample set**									
*FOS*	rs4645869	(S03)	G/A	435/148/17	420/161/16 §	0.1517	0.1524	0.8802	0.7063
	rs4645874	(S04)	C/T	459/128/13	448/145/7	0.1283	0.1325	0.2848	0.9602
	rs17103109	(S05)	T/G	317/241/42	307/247/46	0.2708	0.2825	0.7629	0.5998
*VIPR2*	rs2071623	(S10)	G/A	280/234/86	251/249/100	0.3383	0.3742	0.0053	0.0799
	**rs2071625**	**(S11)** ‡	A/G	303/248/49	259/254/87	0.2883	0.3567	0.0616	**0.0008** ‡
	rs2730220	(S12)	G/A	508/82/10	477/114/9	0.0850	0.1100	0.4095	0.0409
	rs885863	(S13)	G/A	396/178/26	399/186/15	0.1917	0.1800	0.2683	0.4076

* The tag SNPs are listed sequentially from the 5′ end to the 3′ end of the sense strand of the respective gene, and are also designated in this order as S01, S02, …., etc for the sake of easy referencing.

† Allele 1 is the major allele, and allele 2 the minor allele.

‡ In single-marker analysis, these two SNPs were significant even after correction for multiple comparisons: rs2730220 (S12) (*P*_a_ = 0.0017 and *P*_aemp_ = 0.0110) in the replication sample set; and rs2071625 (S11) ((*P*_a_ = 0.0008 and *P*_aemp_ = 0.0046) in the combined sample set.

§ Three control samples failed to be genotyped for rs4645869 even after repeated trials.

**Figure 1:**
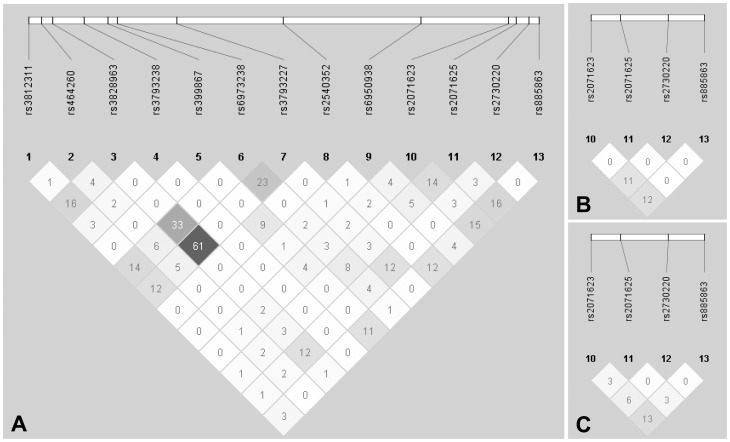
Linkage disequilibrium (LD) patterns for single nucleotide polymorphisms of the *VIPR2* gene. LD measures are indicated as r^2^ values for cases and controls together for (a) the discovery sample set, (B) the replication sample, and (C) the combined sample set. Note that, as defined by solid spine of LD, no LD bock is identified in any of the sample sets.

For candidate genes with two or more tag SNPs examined, we constructed LD patterns with Haploview and defined LD blocks by solid spine of LD. In general, LD between SNPs was very weak with no LD block identified for any of the four genes (*FOS*, *JUN*, *VIP* and *VIPR2*) examined for LD patterns (Figures S1A, S1D and S1E; and Figure 1A). Therefore, we performed haplotype analysis using an exhaustive sliding-window approach (Table 3). Of all the 123 possible sliding windows, 2 windows of the *FOS* gene and 13 windows of the *VIPR2* gene displayed significant association (*P*_aemp_<0.05) with high myopia even after correction for multiple comparisons (n = 123) by permutation test. For the *FOS* gene, the most significant haplotype window was the 3-SNP window S03…S05 consisting of rs4645869, rs4645874 and rs17103109 (*P*_a_ = 6.43×10^−5^ and *P*_aemp_ = 0.0016). For the *VIPR2* gene, the most significant haplotype window was the 4-SNP window S10…S14 made up of rs2071623, rs2071625, rs2730220 and rs885863 (*P*_a_ = 8.47×10^−8^ and *P*_aemp_ = 0.0001). We, therefore, followed up these 7 SNPs by genotyping an independent sample set – the replication sample set (Table 1).

**Table 3:** Summary of exhaustive haplotype analyses based on omnibus tests for sliding windows of all possible sizes across separate sets of tag SNPs of the candidate genes*.

	Sliding Window (SW)		SW with significant omnibus test *P*_aemp_<0.05			The most significant result		
Gene	SNPs/SW	No. of SW	No. of SW	First SW	Last SW	SW†	*P*_a_	*P*_aemp_‡
**Discovery sample set**								
*EGR1*	1	1	0	–	–	S01…S01	0.0816	0.9477
*FOS*	1	5	0	–	–	S03…S03	0.0816	0.9477
	2	4	0	–	–	S03…S04	0.0023	0.0750
	3	3	1	S03…S05	S03…S05	**S03…S05**	**6.43×10^−5^**	**0.0016**
	4	2	1	S02…S05	S02…S05	S02…S05	0.0003	0.0070
	5	1	0	–	–	S01…S05	0.0111	0.3371
*JUN*	1	4	0	–	–	S03…S03	0.4260	1.0000
	2	3	0	–	–	S01…S02	0.0022	0.0722
	3	2	0	–	–	S01…S03	0.0026	0.0845
	4	1	0	–	–	S01…S04	0.0065	0.2124
*VIP*	1	3	0	–	–	S01…S01	0.0237	0.5833
	2	2	0	–	–	S01…S02	0.1110	0.9820
	3	1	0	–	–	S01…S03	0.1720	0.9981
*VIPR2*	1	13	0	–	–	S06…S06	0.0060	0.1950
	2	12	1	S10…S11	S10…S11	S10…S11	1.82×10^−5^	0.0005
	3	11	3	S09…S11	S11…S13	S11…S13	5.93×10^−7^	0.0001
	4	10	2	S09…S12	S10…S13	**S10…S13**	**8.47×10^−8^**	**0.0001**
	5	9	1	S09…S13	S09…S13	S09…S13	3.10×10^−6^	0.0002
	6	8	1	S08…S13	S08…S13	S08…S13	6.87×10^−6^	0.0003
	7	7	2†	S05…S11	S07…S13	S07…S13	9.45×10^−6^	0.0003
	8	6	2	S05…S12	S06…S13	S06…S13	1.05×10^−4^	0.0030
	9	5	1	S05…S13	S05…S13	S05…S13	0.0008	0.0256
	10	4	0	–	–	S03…S12	0.0071	0.2277
	11	3	0	–	–	S03…S13	0.0321	0.6881
	12	2	0	–	–	S02…S13	0.0333	0.7015
	13	1	0	–	–	S01…S13	0.0524	0.8463
**Replication sample set**								
*FOS*	1	3	0	–	–	S05…S05	0.2290	0.8805
	2	2	0	–	–	S04…S05	0.0315	0.2334
	3	1	0	–	–	S03…S05	0.3800	0.9781
*VIPR2*	1	4	1	S12…S12	S12…S12	S12…S12	0.0017	0.0141
	2	3	2	S11…S12	S12…S13	S11…S12	0.0010	0.0076
	3	2	1	S11…S13	S11…S13	S11…S13	0.0032	0.0246
	4	1	1	S10…S13	S10…S13	**S10…S13**	**1.15×10^−5^**	**0.0002**
**Combined sample set**								
*FOS*	1	3	0	–	–	S05…S05	0.6020	0.9998
	2	2	0	–	–	S03…S04	0.2660	0.9184
	3	1	0	–	–	S03…S05	0.1420	0.7269
*VIPR2*	1	4	1	S11…S11	S11…S11	S11…S11	0.0009	0.0095
	2	3	2	S10…S11	S11…S12	S10…S11	7.51×10^−5^	0.0005
	3	2	2	S10…S12	S11…S13	S11…S13	6.51×10^−5^	0.0004
	4	1	1	S10…S13	S10…S13	**S10…S13**	**9.10×10^−10^**	**0.0001**

* The SW is indicated as Sxx…Syy, where Sxx is the first SNP and the Syy the last SNP of the SW. For each candidate gene, the identity of the SNPs (rs numbers) can be found in Table 2. Every SW is assessed by omnibus test adjusted for sex and age to give the *P*_a_ value. For each fixed-size SW, the most significant result is detailed in the last three columns. For the **discovery sample set**, there are a total of 123 SWs across 26 SNPs of the five genes, and multiple comparisons are corrected by running 10,000 permutations to obtain an empirical *P* value that is also adjusted form sex and age (*P*_aemp_). For the **replication sample set** and the **combined sample set**, there are *in each sample set* 16 SWs across 7 SNPs of the two genes tested, and multiple comparisons are corrected by running 10,000 permutations to obtain the *P*_aemp_ values. Note that the minimum *P*_aemp_ value achievable with 10,000 permutations is 0.0001.

† The SW S06…S12 is between S05…S11 and S07…S13, and is not significant for the omnibus test (*P*_aemp_>0.05). Abbreviations: SW, sliding window; *P*_a_, *P* value adjusted for sex and age; and *P*_aemp_, empirical *P* value adjusted for sex and age.

### Replication sample set

We examined seven SNPs with the replication sample set (Table 1) in light of the significant haplotype association obtained with the discovery sample set. We summarize the genotype data of the replication sample set in Table 2. The control group was in HWE for all seven SNPs.

We did not find any significant association in single-marker analysis of *FOS* SNPs, not even when we jointly analyzed both sample sets (hereafter called the combined sample set) (Table 2). However, single-marker analysis of the replication sample set revealed significant association of rs2730220 (S12) of *VIPR2* with high myopia even after correction for multiple testing across seven SNPs (*P*_a_ = 0.0017 and *P*_aemp_ = 0.0110). The OR of its minor allele A was 0.51 (95% CI, 0.34–0.78) with reference to its major allele G. Interestingly, instead of rs2730220 (S12), rs2071625 (S11) of *VIPR2* demonstrated significant association with high myopia for the combined sample sets (*P*_a_ = 0.0008 and *P*_aemp_ = 0.0046) with the OR of its minor allele G being 0.75 (95% CI, 0.63–0.89).

LD between SNPs was also very weak for *FOS* and *VIPR2* in both the replication sample set and the combined sample set (Figures S1B and S1C; and Figures 1B and 1C). We also did not identify any LD block for these sample sets. Exhaustive sliding-window haplotype analysis failed to demonstrate any significant association for *FOS* for both sample sets (Table 3). However, we were able to replicate significant haplotype association (*P*_aemp_<0.05) of *VIPR2* with high myopia: five significant haplotype windows for the replication sample set and six significant haplotype windows for the combined sample set (Table 3). Most importantly, the same *most* significant *VIPR2* haplotype window was identified as in the discovery sample set: S10…S13 consisting of rs2071623, rs2071625, rs2730220 and rs885863 (*P*_a_ = 1.15×10^−5^ and *P*_aemp_ = 0.0002 for the replication sample set; and *P*_a_ = 9.10×10^−10^ and *P*_aemp_ = 0.0001 for the combined sample set).

### The most significant *VIPR2* haplotype window

The 4-SNP haplotype window S10…S13 (rs2071623-rs2071625-rs2730220-rs885863) of *VIPR2* was the most significant haplotype sliding window in the discovery sample set, the replication sample set and the combined sample set (Table 3). The constituent haplotypes of this sliding window are shown in Table 4. We did not observe any haplotypes displaying *opposite* directions of association in the discovery and the replication sample sets although the GAGA haplotype was found in the replication sample set, but not in the discovery sample set. Therefore, the directions of association of these 4-SNP haplotypes were compatible between the discovery and the replication sample sets.

**Table 4:** The most significant *VIPR2* haplotype window in all sample sets: S10…S13 (rs2071623- rs2071625-rs2730220-rs885863) and the constituent haplotypes*.

	Discovery Sample Set					Replication Sample Set					Combined Sample Set				
	Haplotype frequency					Haplotype frequency					Haplotype frequency				
Haplotypes	Cases	Controls	OR	*P*_a_	*P*_aemp_	Cases	Controls	OR	*P*_a_	*P*_aemp_	Cases	Controls	OR	*P*_a_	*P*_aemp_
Omnibus	-	-	-	8.47×10^−8^	0.0001	-	-	-	1.15×10^−5^	0.0002	-	-	-	9.10×10^−10^	0.0001
**GGGG (1211)**	**0.0298**	**0.1097**	**0.18**	**1.85×10^−6^**	**0.0002**	**0.1764**	**0.2221**	**0.72**	**0.0383**	**0.0600**	**0.0982**	**0.1608**	**0.52**	**3.37×10^−6^**	**0.0002**
**GAGA (1112)**	**-**	**-**	**-**	**-**	**-**	**0.0733**	**0.0164**	**14.40**	**1.30×10^−6^**	**0.0001**	**0.0368**	**0.0145**	**4.68**	**0.0001**	**0.0027**
GAGG (1111)	0.5510	0.4448	1.40	0.0032	0.4415	0.3812	0.3746	1.06	0.6250	1.0000	0.4707	0.4132	1.27	0.0049	0.1297
AAGA (2112)	0.0163	0.0641	0.14	7.15×10^−5^	0.0082	0.0734	0.0843	0.82	0.4290	1.0000	0.0475	0.0698	0.55	0.0055	0.1430
GGGA (1212)	0.0281	0.0299	1.00	0.9940	1.0000	0.0273	0.0050	82.50	0.0011	0.0251	0.0309	0.0164	2.54	0.0083	0.2048
AGAG (2221)	0.0352	0.0501	0.63	0.1600	1.0000	0.0085	0.0200	0.16	0.0271	0.4754	0.0238	0.0385	0.53	0.0272	0.4993

* Only haplotypes with *P*_a_<0.05 in the combined sample set are listed here and in the order of increasing *P*_a_ values. Haplotypes and their corresponding statistics are indicated in **boldface** if their *P*_aemp_ values are <0.05 in the combined sample set. Note that the OR of a haplotype is calculated with reference to the remaining haplotypes. This means that the reference haplotypes are different for different haplotypes. In addition, PLINK calculates the OR of a given haplotype without providing the 95% confidence intervals. Abbreviations: OR, odds ratio; *P*_a_, *P* value adjusted for sex and age, and also for sample set for the combined sample set; *P*_aemp_, empirical *P*_a_ value (corrected for multiple testing by permutation test); 1, major allele; and 2, minor allele.

In the combined sample set, GGGG (1211) was a protective haplotype with an OR of 0.52 (*P*_a_ = 3.37×10^−6^ and *P*_aemp_ = 0.0002) and GAGA (1112) a high-risk haplotype with an OR of 4.68 (*P*_a_ = 0.0001 and *P*_aemp_ = 0.0027). Note that the haplotypes are indicated in both the ACGT and the 1–2 (major-minor) formats. The GGGG (1211) haplotype had a frequency of 9.82% in cases and 16.08% in controls. The GAGA (1112) haplotype was much less common with a frequency of 3.68% in cases and 1.45% in controls.

## Discussion

Previous studies indicate that genes responsive to visual signals are involved in the early part of the biological pathways concerned with altered ocular growth while genes responsible for ECM remodeling of the sclera participate in the later part of the pathways [29]–[31]. Therefore, we selected *EGR1*, *FOS*, *JUN*, *VIP* and *VIPR2* as functional candidates and investigated their potential association with high myopia. We assumed a “common disease common variants” model [43] in this study and hence selected SNPs, the most abundant sequence variation in the human genome, as the genetic markers for the present genetic association study.

We performed haplotype analysis in addition to single-marker analysis. In the absence of LD block defined for the genes under study (Figures S1 and 1), we used the variable-sized sliding-window strategy to further explore possible association by comprehensively examining haplotype windows of all possible sizes. This strategy has been shown to be more powerful in detecting genetic association than single-marker analysis and LD-block-based haplotype analysis [44]. This is particularly true for genomic regions of low LD such as candidate loci evaluated in this study (Figures S1 and 1).

We did not find any evidence to support the role of *ERG1*, *FOS*, *JUN* and *VIP* in the genetic susceptibility to high myopia (Tables 2 and 3). We failed to confirm with the replication sample set the initial significant association of *FOS* haploypes with high myopia in the discovery sample set. Our *EGR1* data complement a recent study that assumed a “common disease rare variants” model and did not find any pathological mutation in the *EGR1* coding regions by DNA sequencing of 96 Chinese subjects with high myopia [45].

On the contrary, we first found the significant association of *VIPR2* haplotypes with high myopia in the discovery sample set and then successfully replicated the significant association in the replication sample set (Tables 2 and 3). We found it reassuring that the haplotype window S10…S13 (rs2071623-rs2071625-rs2730220-rs885863) was the most significant sliding window among all possible haplotype windows examined in the discovery, the replication and the combined sample sets (Table 3). Although the haplotype GAGA (1112) of the S10…S13 window was significant in the replication sample set, it was not found in the discovery sample set (Table 4). Other than this, the directions of association were consistent for the haplotypes identified in the discovery and the replication sample sets. In the combined sample set, we identified one protective haplotype (GGGG or 1211, OR = 0.52) and one high-risk haplotype (GAGA or 1112, OR = 4.68). The high-risk haplotype GAGA was much less common particularly in the controls: 1.64% (∼20 chromosomes out of 1200 chromosomes) in the discovery sample set and 1.45% (∼17 chromosomes out of 1200 chromosomes) in the replication sample set. Note that analysis for this rare haplotype might be subject to random variation.

In the combined sample set, the four constituent SNPs of the *VIPR2* S10…S13 window each contributed independent effects to the significant haplotype association as shown by conditional logistic regression: *P* = 5.37×10^−8^ for S10 (rs2071623), *P* = 4.31×10^−7^ for S11 (rs2071625), *P* = 0.0181 for S12 (rs2730220) and *P* = 1.09×10^−8^ for S13 (rs885863). We note that these four SNPs are located at the 3′ end of the *VIPR2* gene (Figure S2). This region harbors a few sequence features that may be important in regulating the expression of *VIPR2*.

The importance of S11 (rs2071625) was highlighted by its significant association in single-marker analysis (Table 2) and its being a constituent SNP in all significant haplotype windows (Table 3) in the combined sample set. In the discovery sample set, S11 (rs2071625) was the only SNP included in all 13 significant haplotype windows (Table 3) even though it was not associated with high myopia as a single marker (Table 2). On the other hand, S12 (rs2730220) stood out in the replication sample set because of its significant association as a single marker. In both scenarios, association was statistically more significant with haplotype windows than with single markers. Therefore, we speculate that these SNPs or their haplotypes are more likely tagging some untyped causal variants that drive the genuine association with high myopia. Nevertheless, some databases (e.g., Patrocles, http://www.patrocles.org/Patrocles_targets.htm; and FuncPred, http://manticore.niehs.nih.gov/snpinfo/snpfunc.htm) predict that S12 (rs2730220) and S13 (rs885863) may affect the binding of certain microRNAs and hence influence the expression of *VIPR2* accordingly. Despite these predictions, we doubt the involvement of these SNPs as *causal* variants in the genetic susceptibility to high myopia. Therefore, we recommend that future studies be aimed at identifying the causal variants. Since this is also the first study that has identified *VIPR2* as a myopia susceptibility gene, our positive results should be replicated using samples from other populations, particularly those of different ethnicities.

*VIPR2*, also known as *VPAC2*, is located on chromosome 7q36 and lies within a putative locus for autosomal dominant high myopia (once called *MYP4*) [37], [38]. As a G-protein coupled receptor, VIPR2 is in fact a receptor for VIP. The expression of VIPR2 in the retina and the choroid was up-regulated in the treated eyes with reference to the fellow control eyes, but down-regulated with increasing axial length in chicks with form-deprivation myopia [39]. An unselective antagonist of VIP receptors (including VIPR2) could also suppress the development of form-deprivation myopia in chicks in a dose-dependent manner [46]. *VIPR2* is also an input gene involved in circadian networks. Intriguingly, some studies have shown that transgenic mice over-expressing or lacking VIPR2 show deranged circadian rhythms [47]–[49]. Interestingly, growing eyes of chicks and monkeys display a diurnal rhythm in axial length, which is in anti-phase with the rhythm in choroidal thickness [50]. These phases are distorted in eyes that grow too fast or too slowly. Therefore, we speculate that VIPR2 may influence genetic susceptibility to myopia through its involvement in circadian rhythms. In light of the significant association between *VIPR2* gene polymorphisms with high myopia, this hypothesis is worth exploring in future studies.

In summary, *EGR1*, *JUN*, *FOS* and *VIP* were not associated with high myopia. However, we identified *VIPR2* as a novel myopia susceptibility gene. We obtained consistent results with sliding-window haplotype analysis and the S10…S13 haplotype window (rs2071623-rs2071625-rs2730220-rs885863) was the most significant sliding window in all sample sets. In combined sample set, S11 (rs2071625) also showed significant association with high myopia as a single marker.

## Supporting Information

Figure S1 **Linkage disequilibrium (LD) patterns for single nucleotide polymorphisms of *FOS*, *JUN* and *VIP*.**
(PDF)

Figure S2 **Sequence features at the 3′ end of the *VIPR2* locus.**
(PDF)

Table S1 **SNP genotyping: restriction enzymes, and sequences of primers, extension primer, and probes used.**
(DOC)

Appendix S1 **Genotyping of single nucleotide polymorphisms (SNPs).**
(DOC)
