# Supplementary material for: Genetic Susceptibility to Refractive Error: Association of Vasoactive Intestinal Peptide Receptor 2 (VIPR2) with High Myopia in Chinese
Source: PLoS One. 2013 Apr 18;8(4):e61805. doi: 10.1371/journal.pone.0061805 (PMC3630195; doi:10.1371/journal.pone.0061805)
Supplement: Figure S1 — Linkage disequilibrium (LD) patterns for single nucleotide polymorphisms of FOS, JUN and VIP. (PDF) [file pone.0061805.s001.pdf]

Genetic susceptibility to refractive error: association of vasoactive intestinal peptide receptor 2 (*VIPR2*) with high myopia in Chinese
Yiu WC, Yap MH, Fung WY, Ng PW, Yip SP

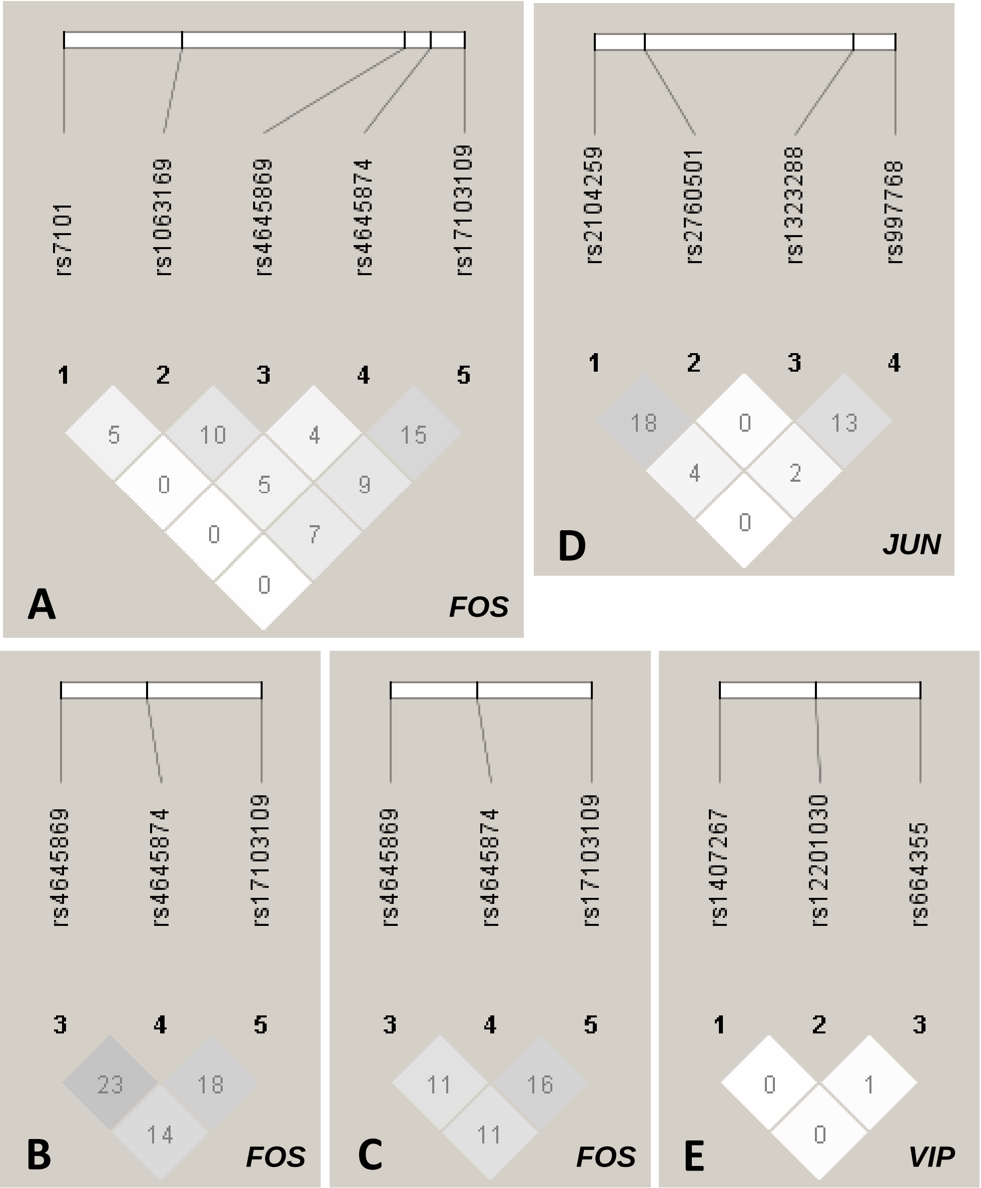

Figure S1. Linkage disequilibrium (LD) patterns for single nucleotide polymorphisms of *FOS, JUN* and *VIP*.

LD measures are indicated as r^2 values for cases and controls together. The LD patterns of the *FOS* gene are shown for (A) the discovery sample set, (B) the replication sample set, and (C) the combined sample set. The LD patterns of (D) the *JUN* gene and the (E) the *VIP* gene are shown for the discovery sample set. Note that, as defined by solid spine of LD, no LD block is identified in any of the sample sets for any one of the three genes.
