# Supplementary material for: Genetic Susceptibility to Refractive Error: Association of Vasoactive Intestinal Peptide Receptor 2 (VIPR2) with High Myopia in Chinese
Source: PLoS One. 2013 Apr 18;8(4):e61805. doi: 10.1371/journal.pone.0061805 (PMC3630195; doi:10.1371/journal.pone.0061805)
Supplement: Figure S2 — Sequence features at the 3′ end of the VIPR2 locus. (PDF) [file pone.0061805.s002.pdf]

Genetic susceptibility to refractive error: association of vasoactive intestinal peptide receptor 2 (*VIPR2*) with high myopia in Chinese
Yiu WC, Yap MH, Fung WY, Ng PW, Yip SP

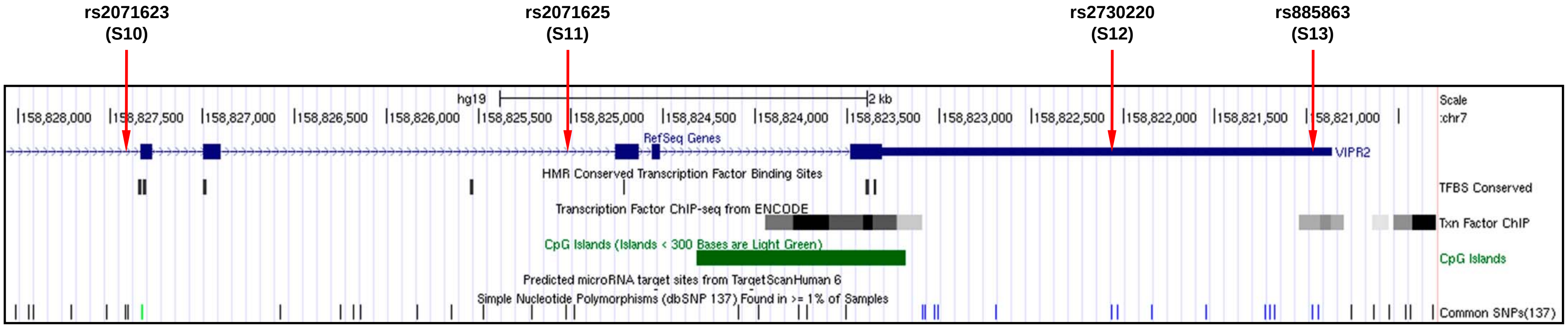

Figure S2. Sequence features at the 3' end of the *VIPR2* locus.
Below the base position on chromosome 7 based on GRCh37/hg19 assembly shows the exons (blue boxes) and introns (thin lines with arrows) at the 3' end of the *VIPR2* gene. Red arrows show the positions of the four single nucleotide polymorphisms (SNPs) that form the most significant haplotype windows in all sample set. Next panel shows several putative transcription factor binding sites predicted by aligning human/mouse/rat (HMR) sequences. The Txn Factor ChIP track shows DNA regions where transcription factors bind as assayed by chromatin immunoprecipitation with antibodies specific to the transcription factor followed by sequencing of the precipitated DNA (ChIP-seq). Shown in green are predicted intragenic CpG islands. No binding site for microRNA is predicted by TargetScan version 6. The bottom track shows the locations of common single nucleotide polymorphisms (SNPs). These sequence features are captured with the UCSC genome browser. (Available online only)
