# Supplementary material for: Genetic Susceptibility to Refractive Error: Association of Vasoactive Intestinal Peptide Receptor 2 (VIPR2) with High Myopia in Chinese
Source: PLoS One. 2013 Apr 18;8(4):e61805. doi: 10.1371/journal.pone.0061805 (PMC3630195; doi:10.1371/journal.pone.0061805)
Supplement: Table S1 — SNP genotyping: restriction enzymes, and sequences of primers, extension primer, and probes used. (DOC) [file pone.0061805.s003.doc]

Table S1. SNP genotyping: restriction enzymes, and sequences of primers, extension primer, and probes used

| Gene | SNP * | Genotyping method | Primer / Probe Sequence (5’ to 3’) |
| --- | --- | --- | --- |
| *EGR1* | rs11741807 (S01) | RFLP : MvaI | F1: GCC TGT TCC TCT TCA GTC TGT  R1: CTT GAG AAC TGG CCT TTA CCT C |
|  |  |  |  |
| *FOS* | rs7101 (S01) | MC : Excess primer F1  : 1.5 μM Syto 9 | F1: CGT ACT CCA ACC GCA TCT G  R1: GAG TGG TAG TAA GAG AGG CTA T  P1: CGC TGT GAA GCA GAG ATG GGT AGG A - Phos |
|  | rs1063169 (S02) | RFLP : PsyI | F1: (T)_24_TGA GGA TCT TAT TTT AAA TGC AAG TCA GAC C  R1: (T)_8_ CAA AAA AAG ACC GAC ATT CAG TGA CAG T |
|  | rs4645869 (S03) | MC : Excess primer F1  : 1.5 μM Syto 9 | F1: GAG AGG CTG AGA CAG GAA A  R1: CCA TTA TTT GTG CTG TTT GAG ATA T  P1: GGT ATT AGA GAA ACC AGA AAC CAC AAT GC - Phos |
|  | rs4645874 (S04) | MC : Excess primer R1  : 1.5 μM Syto 9 | F1: GCT TTA ACT TAG AAC TTT ATC ATC TTA A  R1: GCA CCC AGT GAT TTT CAT TAT C  P1: CGA GTA TCA CGC AGG CAC CAG GC - Phos |
|  | rs17103109 (S05) | RFLP : BsaJI | F1: GTT TCT GCT AGA CTT GTG ATG ACA  R1: CCT GCC TCA GCC TCC TG |
|  |  |  |  |
| *JUN* | rs2104259 (S01) | RFLP : HinfI | F1: (T)_25_ TGTT ACA TTC CTT TGT ATT TTC TCG ACT  R1: (T)_7_ GAA CGG AGTT ACA ATC AGA GAA TC |
|  | rs2760501 (S02) | MC : Excess primer R1  : 1.5 μM Syto 9 | F1: CCC TGT CAC ACT AAC TCC  R1: ATG TAA GTG GTT CCT TTT CCG  P1: TTT GCT TGC TTC ACA CTG TGG TCG CTT G - Phos |
|  | rs1323288 (S03) | RFLP : HinfI | F1: TTG CTT TTG CTG CCT GAT AAT CAC  R1: CAA ATA AAC CCC TAT GCC TAT GGA |
|  | rs997768 (S04) | PE-DHPLC | F1: GTG AAA GAG CAT GGG ATT TAG AT  R1: GGA CAT TTA ACC CTC TCT GTC TAT CAA GAC  PE1: ATG GAA CTC TGG GAG GGT C |
|  |  |  |  |
| *VIP* | rs1407267 (S01) | MC : Excess primer F1  : 1.5 μM Syto 9 | F1: GTA AAT GCT AAG TGC TTG CCT  R1: TAC CTG ATT ATT ACA AAC TAG AGG  P1: ATT GAG GTT CAC AGA CAA ATG ACT GCT GA - Phos |
|  | rs12201030 (S02) | RFLP : MboI | F1: CAT AAT ATC ATA TTC ATT GAC TCC AGA C  R1: TTT AGA AGC TAT GCT ACC CTG ATC A |
|  | rs664355 (S03) | RFLP : HincII | F1: (T)_16_ AAA TAG GAG AAA AAT TGT TGA CCA TGT CA  R1: (T)_28_ AAT CTA CCA TGC AGC AGG AGT TG |
|  |  |  |  |
| *VIPR2* | rs3812311 (S01) | RFLP : BseGI | F1: GTT CGT GGT CCT CAG CGG AT  R1: CCT ACT TAT ATC AGA AAG AAA ATG AAA AGG AAA |
|  | rs464260 (S02) | RFLP : NspI | F1: ACT GGA ACC TGG ACT GGG AT  R1: (T)_16_ TTC CGT GAA TGA TTG CAT GTG GAA T |
|  | rs3828963 (S03) | RFLP : MboI | F1: CTT GGA GAG GGT GAA CAT TCA AG  R1: GCA CAC AGG TGG GAG GC |
|  | rs3793238 (S04) | RFLP : MboII | F1: CCA TAC AGA CCA CAC CTT CAG  R1: TGT CAG AGC GTT GGG GTT GT |
|  | rs399867 (S05) | MC : Excess primer R1  : 2.0 μM Syto 9 | F1: CGA GTG GGA AAA GGA AAG  R1: AGG AAG AGA GTG GCT CAT  P1: TAG AAG CAA ATA TGT CCA ATC GCA TCC TC -Phos |
|  | rs6973238 (S06) | RFLP : Alw26I | F1: GGT TTG GTT CCT CAG TTG CAG  R1: CGT GAT CTG AAA AGG CCA AGG |
|  | rs3793227 (S07) | RFLP : AluI | F1: TGG TGG GGG CAG AAG ACA TT  R1: GCT CTA CAC TGA CAG ACT CCA |
|  | rs2540352 (S08) | MC : Excess primer F1  : 2.0 μM Syto 9 | F1: AGG TGA GAA GGT CCC ATC  R1: GTT CTA AAC ATT GGT GCT TAT TAC  P1: ACA GTA GCG TGG CAG TTT TCT TGT GAG TT - Phos |
|  | rs6950938 (S09) | MC : Excess primer R1  : 2.0 μM Syto 9 | F1: GAG GTT GGA GTG TTG GAG GA  R1: GAC CAG TGC TTA GGA AGT GC  P1: GCT TTC TTC TGT GGA CAT CGC TGT GCC - Phos |
|  | rs2071623 (S10) | MC : Excess primer R1  : 2.0 μM Syto 9 | F1: GGA CTA CTT ACG ATG ATG GAA  R1: AGC AGA CTA TTG TTT TGA AAG CA  P1: CAA GTT GGG AGG GGC AGG CGT TCA TTC - Phos |
|  | rs2071625 (S11) | RFLP : NspI | F1: TCT TGT CAC ATG CCT GCC TCA  R1: CTC AAC ACC TCC TCT CCA CT |
|  | rs2730220 (S12) | RFLP : Bg1I | F1: (T)_24_ TGT CTG GGG ACG TTG CCG AG  R1: TGA GGT CCA TCG AAC GCC GTG |
|  | rs885863 (S13) | RFLP : HincII | F1: CCA AGT GTG TCT TCC ATG CTC  R1: GTT CAG CGG AGT TGA CTG GTT |

* The tag SNPs are listed sequentially from the 5’ end to the 3’ end of the sense strand of the respective gene, and are also designated in this order as S01, S02, …., etc for the sake of easy referencing.

Abbreviations: RFLP, restriction fragment length polymorphism; MC, unlabeled probe melting curve analysis; PE-DHPLC, primer extension reaction coupled with denaturing high performance liquid chromatography; F1, forward primer; R1, reverse primer; PE1, primer for extension; and P1, probe with 3’ phosphate (Phos).
