# Supplementary material for: Genetic Susceptibility to Refractive Error: Association of Vasoactive Intestinal Peptide Receptor 2 (VIPR2) with High Myopia in Chinese
Source: PLoS One. 2013 Apr 18;8(4):e61805. doi: 10.1371/journal.pone.0061805 (PMC3630195; doi:10.1371/journal.pone.0061805)
Supplement: Appendix S1 — Genotyping of single nucleotide polymorphisms (SNPs). (DOC) [file pone.0061805.s004.doc]

**Appendix S1**

**Genotyping of single nucleotide polymorphisms (SNPs)**

**SNP genotyping by restriction fragment length polymorphism (RFLP)**

Sixteen SNPs were genotyped by this method as shown in Table 1 (available at [http://plosone.org](http://aaojournal.org/)). Polymerase chain reaction (PCR) was performed in a 10-µL reaction mixture containing 10 ng genomic DNA, 2.5 mM MgCl_2_, 0.1 µM each of the forward and reverse primers (Table 1, available at [http://plosone.org](http://aaojournal.org/)), 0.2 mM of each dNTP and 1×  Gold Buffer, and 0.2 U of Ampli*Taq* Gold DNA polymerase (Applied Biosystems, Foster City, California). Amplification was performed in 96-well plates with a thermocycler (GeneAmp 9700 PCR System; Applied Biosystems), including 1 cycle of initial denaturation at 95°C for 5 minutes, 35 cycles of 95°C for 30 seconds, 58°C for 45 seconds and 72°C for 30 seconds, and an additional extension cycle at 72°C for 5 minutes. Specific restriction enzymes (MBI Fermentas, Vilnus, Lithuania; Table 1, available at [http://plosone.org](http://aaojournal.org/)) were added to the PCR products according to the manufacturer’s recommendations. Digested products were separated by electrophoresis in polyacrylamide gels of appropriate concentration. An internal control restriction site was always included in the PCR products for all SNPs genotyped by this method to avoid genotyping errors due to incomplete or failed digestion.

**SNP genotyping by unlabelled probe melting curve analysis**

Nine SNPs were genotyped by this method as shown in Table 1 (available at [http://plosone.org](http://aaojournal.org/)). Asymmetric PCR was performed as described above, but with changes in the primer ratio, DNA polymerase and the annealing temperature. The final concentration was 0.1 µM for the excess primer, and 0.01 µM for the limiting primer (Table 1, available at [http://plosone.org](http://aaojournal.org/)). HotStar Taq Plus DNA polymerase (2 U; Qiagen, Hidlen, Germany) was used together with the 1× PCR buffer (with KCl and (NH4)_2_SO_4_) for PCR reaction. In order to increase the amount of single-stranded PCR product, the annealing temperature was set at 55°C, and both annealing and extension time were extended to 60 seconds with 50 cycles. After amplification, a 10-µl melting mixture containing PCR products, 0.5 µM unlabeled probe and 1.5 or 2.0 µM Syto 9 (Invitrogen, Carlsbad, CA; Table 1, available at [http://plosone.org](http://aaojournal.org/)) was prepared. Melting curve analysis was performed on a LightCycler 480 PCR System (Roche Applied Systems, Basel, Switzerland). The melting data were collected between 50°C and 95°C at 5 acquisitions per °C, using the “melting curves genotyping” analysis mode.

**SNP genotyping by primer extension coupled with denaturing high-performance liquid chromatography (PE-DHPLC)**

One SNP (rs997768; S04 of *JUN*) was genotyped by this method (Table 1, available at [http://plosone.org](http://aaojournal.org/)). PCR was performed as described above for RFLP. PCR products (10 µL) were purified with 5 U of exonuclease I (New England Biolabs, Beverly, MA) and 0.5 U of shrimp alkaline phosphatase (GE Healthcare, Piscataway, NJ) at 37°C for 30 minutes, and the enzymes were then inactivated at 80°C for 20 minutes. The purified PCR products (8 µL) were then used as templates in a 25-µL primer extension reaction mixture, which included 50 µM each of ddATP, ddGTP and ddTTP (GE Healthcare), and 1U Therminator (New England BioLab), 1× reaction buffer (26 mM Tris-HCl, 6.5 mM MgCl_2_, pH 8.5) and 0.6 µM extension primer (Table 1, available at [http://plosone.org](http://aaojournal.org/)). Amplification was performed as follows: 1 cycle of initial denaturation at 96°C for 1 minute, 55 cycles of 96°C for 10 seconds, 43°C for 15 seconds and 60°C for 1 minute. The reaction mixture was then analyzed with the WAVE DNA fragment analysis system (Transgenomic, Omaha, NE).
